# Calibration and Cross-Validation of Accelerometery for Estimating Movement Skills in Children Aged 8–12 Years

**DOI:** 10.3390/s20102776

**Published:** 2020-05-13

**Authors:** Michael J. Duncan, Alexandra Dobell, Mark Noon, Cain C. T. Clark, Clare M. P. Roscoe, Mark A. Faghy, David Stodden, Ryan Sacko, Emma L. J. Eyre

**Affiliations:** 1 Centre for Sport, Exercise and Life Sciences, Coventry University, Coventry CV1 5FB, UK; ad0183@coventry.ac.uk (C.C.T.C.); ab2223@coventry.ac.uk (E.L.J.E.); 2 School of Human Sciences, University of Derby, Derby DE22 1GB, UK; a.dobell1@unimail.derby.ac.uk (A.D.); aa5349@coventry.ac.uk (M.N.); c.roscoe@derby.ac.uk (C.M.P.R.); m.faghy@derby.ac.uk (M.A.F.); 3 Department of Physical Education, College of Education, University of South Carolina, Columbia, SC 29208, USA; stodden@mailbox.sc.edu; 4 Department of Health and Human Performance, The Citadel, Charleston, SC 29409, USA; rsacko@citadel.edu

**Keywords:** motor competence, motor development, indirect calorimetry, energy expenditure, wearables, sensors

## Abstract

(1) Background: This study sought to calibrate triaxial accelerometery, worn on both wrists, waist and both ankles, during children’s physical activity (PA), with particular attention to object control motor skills performed at a fast and slow cadence, and to cross-validate the accelerometer cut-points derived from the calibration using an independent dataset. (2) Methods: Twenty boys (10.1 ±1.5 years) undertook seven, five-minute bouts of activity lying supine, standing, running (4.5kmph^−1^) instep passing a football (fast and slow cadence), dribbling a football (fast and slow cadence), whilst wearing five GENEActiv accelerometers on their non-dominant and dominant wrists and ankles and waist. VO_2_ was assessed concurrently using indirect calorimetry. ROC curve analysis was used to generate cut-points representing sedentary, light and moderate PA. The cut-points were then cross-validated using independent data from 30 children (9.4 ± 1.4 years), who had undertaken similar activities whilst wearing accelerometers and being assessed for VO_2_. (3) Results: GENEActiv monitors were able to discriminate sedentary activity to an excellent level irrespective of wear location. For moderate PA, discrimination of activity was considered good for monitors placed on the dominant wrist, waist, non-dominant and dominant ankles but fair for the non-dominant wrist. Applying the cut-points to the cross-validation sample indicated that cut-points validated in the calibration were able to successfully discriminate sedentary behaviour and moderate PA to an excellent standard and light PA to a fair standard. (4) Conclusions: Cut-points derived from this calibration demonstrate an excellent ability to discriminate children’s sedentary behaviour and moderate intensity PA comprising motor skill activity.

## 1. Introduction

The importance of physical activity (PA) for children’s health is well established [1], and considerable efforts to encourage children and youth to engage in PA via different forms of intervention are evident in the scientific literature [1]. The World Health Organisation advocate that children, aged 5–17 years, should accumulate at least 60 min moderate-to vigorous intensity PA daily [2]. However, data from 122 countries worldwide show that over 80% of children fail to achieve this guideline [1]. An inherent issue when exploring PA levels in children relates to the methods of assessing PA, as, without an accurate means to measure PA, understanding how many children meet the aforementioned PA guidelines efforts to improve PA may be less effective. Importantly, the types of PA suggested to achieve recommended PA guidelines arise from different forms of movement e.g., sports, games and leisure activities, which encompass a multitude of movements that are both continuous (e.g., walking or running) and discrete (e.g., kicking, throwing, striking) in various forms [3]. Such activities also take place under different constraints and in different environments [4,5] with Newell’s model [6] suggesting that task, individual and environmental constraints interact in the development of movement. Collectively, this poses a challenge to the assessment of children’s PA, which is recognised as being more sporadic when compared with adults [7] and, in line with Newell’s model, accurate assessment of PA must be sensitive to individual constraints (e.g., body size), environmental constraints (e.g., different types of surfaces) and task constraints (e.g., the goal of the task or different types of equipment) [6].

The use of accelerometery has been positioned as the most effective means to objectively assess PA and sedentary behaviour (SB) in children, as it provides a measure which is more reliable and valid when compared to alternative methods, such as self-report or direct observation [7]. Likewise, although objective measures such as pedometry do provide a quantification of the total volume of PA undertaken in steps/day and are relatively cheap to purchase, they are not sensitive to non-linear movement, and are unable to provide a quantification of the amount of time spent in different intensities of PA [7]. Despite the relative expense of accelerometers and the more complex data processing and handling demands, the use of accelerometers for paediatric-based PA research has also grown over the last decade [8,9]. Considerable efforts have also been made to calibrate accelerometer cut-points to more accurately classify PA and SB in paediatric populations [10,11]. There do, however, remain limitations in the scientific understanding of the application of accelerometery, particularly in children and youth. Notably, classification of PA has to date been largely dependent on selection of cut-points from studies that mainly involve continuous locomotion and the choice of accelerometer placement (e.g., hip, wrist) [12].

These two aforementioned points are crucial when considering the effectiveness of accelerometers in assessing children’s PA. Although the cut-points are available to classify accelerometer derived PA in children [10,11,12], the majority of these rely primarily on activities forwards ambulatory movement, which is likely to limit their accuracy when applied to a movement that is multidirectional. Likewise, while the wrist and hip are the predominant placement locations used in prior work [10,11,12], such placement locations may be less accurate when assessing activities requiring multi-directional lower limb movement, such as those commonly encountered in team sports such as football or rugby. Such issues have been highlighted by previous reviews in the area [13]. As a consequence, there remains a need to improve accelerometer studies to better refine the accuracy of such measures to assess children’s PA and SB. The accuracy of accelerometery in tasks requiring greater use of the upper body, cycling, or non-linear movement has been questioned [7], and recently there has been a recognition that classification of fundamental motor skills (FMS) needs to be considered in the context of accelerometer-based PA assessment in children [14,15]. In particular, FMS, such as kicking and throwing, are conceptualised as important and a regular feature of children’s PA [16], and the classification of these types of movement is a noted limitation of accelerometery [13]. For example, while throwing may result in a PA intensity that is moderate in nature, an accelerometer placed on the wrist may be able to more accurately assess this activity than, say, an accelerometer on the waist or ankle. Likewise, in passing a football an accelerometer placed on the ankle may be better able to assess the intensity of PA compared to say one placed on the wrist or waist. This issue is compounded if cut-points are used that have not been calibrated against similar types of activities. If the intention is to capture whole day, or multi day PA, where motor skill type activities feature, it is important that accelerometery accurately classifies the intensities of different types of activity that children engage in, including FMS. Examining how accelerometers perform in assessing different types of FMS is a current need, given the most recent UK guidelines for PA [17] emphasise that children should engage in PA to develop movement skills, due to its reciprocal association with PA.

One recent study by Duncan et al. [14] compared indirect calorimetry-assessed PA with accelerometer derived estimates on the dominant and non-dominant wrist, waist and dominant ankle. Children undertook a protocol comprising locomotor (walking, running, cycling) and object control (throwing and catching, passing a football) activities. Their results suggested that accelerometers, regardless of placement location, classified SB excellently, but the discrimination of activity intensity was considered ‘good’ for moderate PA for waist and ankle location, but ‘fair’ for either wrist location [14]. The authors also concluded that ankle placement provides the most suitable wear location to assess children’s PA intensity, where FMS activities are included.

The study by Duncan et al. [14] remains the only study to date to examine PA during FMS using an ankle placed accelerometer. As a consequence, additional work is needed to substantiate the suggestion on ankle-based accelerometer placement being superior to any other placement location. Placement location is a key consideration when using accelerometers to assess PA [18] and the ankle is only recently being cited in the literature [8]. Despite these results suggesting an ankle placement location may offer greater accuracy in assessing PA, the feasibility, practicality and social desirability of wearing a monitoring device on the ankle over other more commonly worn locations, such as the waist and hip needs to be considered. Likewise, it is not clear whether an ankle placement is more effective in capturing PA intensity during accelerometery, compared to other placement locations. The waist has been the most commonly used placement site when employing accelerometers to assess PA [19], as, historically, studies in which accelerometers were affixed at different anatomical sites, vertical acceleration at the hip had the highest correlation coefficient with measured energy expenditure (EE) during common physical activities, such as walking and stepping [20], and is closest to the centre of mass [21]. Additionally, in more contemporary work, waist placement tends to perform better than wrist-worn monitors at characterising PA intensities [9]. However, despite the ubiquity of waist-affixed monitors, studies have tended to report higher compliance rates with wrist-worn accelerometers, especially in younger children [9], and they facilitate longer wear time for longitudinal monitoring studies [22]. Prior studies with adult participants [5] have reported that ankle derived PA is similar, or better than, waist or wrist worn locations for estimating energy expenditure, and Crouter et al. [8] reported that energy expenditure, estimated from an ankle worn actigraph, was comparable to that determined via indirect calorimetry in a sample of 8–15-year-olds. Furthermore, Clark et al. [23] noted that no, singular, accelerometer wear site ‘fits all’, explicating that current evidence, albeit in adults, suggests an ankle worn monitor may be the most efficacious locale for activity classification. Indeed, to date, only Duncan et al. [14] have compared ankle worn accelerometer placement to other wear sites when classifying PA and SB in children. While the aforementioned study did include an assessment of some FMS in its protocol, the activities were restricted to throwing and catching and instep passing a football (soccer ball) at a set cadence. Recent work has identified the cadence of discrete skill performance as a significant influence on the level of PA attained [15,24]. Specifically, the cadence, or rate at which a discrete skill is performed, usually quantified as repetitions per minute, can influence the subsequent intensity of that activity, or the level of energy expended during that activity [14,15,24].

The current study, therefore, sought to address gaps in the literature base relating to accelerometer derived PA by: (a) examining the energy expenditure during object control skills in children performed at fast and slow cadence, as related to sedentary activity and running; (b) calibrating triaxial accelerometery (i.e., determining accelerometer cut-points), worn on both wrists, waist and both ankles, during children’s PA, with particular attention to object control FMS performed at fast and slow cadence and; (c) cross-validating the accelerometer cut-points derived from the accelerometer calibration using an independently collected dataset.

## 2. Materials and Methods

### 2.1. Participants

In order to calibrate the accelerometers, a convenience sample of 20 healthy, Caucasian, boys aged between 8 and 12 years of age (10.1 ± 1.5 years) took part in this study, following institutional ethics approval (registration number: P87814), parental written informed consent and child assent. Mean ± SD of height, mass and body mass index (BMI), was 1.4 ± 0.2 m, 36.5 ± 7.5 kg and 17.3 ± 1.9 kg/m^2^, respectively. Given the object control tasks used in the present study, the children that participated were all involved in grassroots junior football (i.e., regularly played league football but not as part of a professional football academy setup) as part of their recreational sports activities.

For cross-validation, the data set employed has been described previously (See [14]). This comprised 30 children (16 boys, 14 girls) aged 8–11 years of age (9.4 ± 1.4 years) recruited from central England, who were also involved in grassroots football as part of their recreational sports activities.

### 2.2. Procedures (Calibration)

For the calibration, participants wore a GENEActiv monitor (Activinsights, Cambridge) on their non-dominant wrist, dominant wrist and dominant waist (defined as the hip of the dominant leg), similar to other work [25], as well as an additional monitor placed on both the dominant ankle (as per [14]) and non-dominant ankle. The dominant ankle was determined by asking the children which leg they considered the leg most used for kicking which was subsequently verified with their parents. The GENEActiv devices were secured at the waist and ankle using purpose built straps made by the manufacturer (Activinsights, Cambridge), while the device, when worn at the wrist, was secured using the regular wrist watch style strap used in prior studies [14]. The GENEActiv has been extensively described in detail previously [26]. It is a lightweight triaxial accelerometer which provides raw acceleration data. It has high intra and inter-instrument reliability (coefficient of variation = 1.8% and 2.4% respectively) and good criterion-referenced validity (r = 0.97) when compared to a multi-axis shaking table and high concurrent validity with the Actigraph GT1M accelerometer [26].

The GENEActiv was chosen as it provides tri-axial raw accelerometery data from monitors that can be worn on multiple body locations. It is also capable of capturing high-frequency data (up to 100 Hz) for multiple days (up to 7 days at 100 Hz or 45 days at 10 Hz), and is thus attractive for researchers interested in assessing PA. In the current study, the GENEActiv was set to record at 80 Hz and 1 s epochs, as per prior work [11]. During testing, indirect calorimetry was employed. $\dot{V}$O_2_ and $\dot{V}$CO_2_ was assessed using a MetaMax 3B (Cortex Biophysik GmbH, Leipzig, Germany) breath by breath gas analyser. Participants wore a junior face mask (Hans Rudolph), and the calorimeter was calibrated with gases of known concentration each day prior to commencing testing.

After briefing, but prior to beginning the protocol, each participant was fully familiarised with the treadmill being used in the study (Woodway Inc, Wisconsin, WI, USA). Once the GENEActiv monitors and gas analyser had been fitted, each participant performed a series of activities reflective of different levels of PA. These were lying supine, standing and a medium-paced run (4.5 kmph^−1^). Participants then performed two bouts (slow and fast cadence) of instep passing a football and two bouts of dribbling a football (slow and fast cadence). For children aged 8–9 years, a size 3 ball was used, and for children aged 10–12 years a size 4 ball was used, in line with recommended ball sizes used for those age groups by the Football Association of England. Instep passing was performed over a distance of 5 metres at a cadence of 1 pass every 6 seconds (10 per min, slow cadence) and 1 pass every 3 s (20 per min, fast cadence). Children were instructed to control and pass the ball with their dominant foot. Passes to each child were given and received by an FA qualified Coach. Football dribbling was performed over a 3-metre course, where the ball was dribbled around 4 cones using both feet at a cadence of 4 cones every 10 s (6 dribbles per min, slow cadence) and 4 cones every 5 s (12 dribbles per min, fast cadence).

All activities were performed for 5 min, with a 5-minute rest in between. Using previous protocols [12] as guidelines. Running speed was 4.5 kmph^−1^ to represent a medium pace [12,14]. Given the predominant basis for accelerometer calibration studies have focused on running and walking activity, the running condition was employed in the current study in a manner akin to a control, to compare the object control skill performance against.

### 2.3. Data Processing

Data processing procedures followed recognised protocols for the use of accelerometer data. These were consistent for the calibration and cross-validation aspects of the present study. Upon completion of the protocol, each participant’s accelerometer and calorimetry data was downloaded and stored on a computer. The first two minutes and last minute of each bout was discarded, leaving a 2-minute period for analysis. Graphical inspection of data was used to verify the activity intensities were at steady state [27,28]. Using the GENEActiv post-processing software (Version 2.9), the raw 80 Hz signal from all three axes were summarised into a single vector magnitude (gravity subtracted) (SVM gs), congruent with prior work by other authors [10,11,14,26]. The correction for gravity was undertaken to focus the outcome variable on dynamic, rather than static accelerations, as recommended by Esliger et al. [26], and used by prior authors [10,11]. Data were saved in a raw format as binary files, and then data for each wear location were summed into a signal magnitude vector (gravity subtracted) expressed in 1s epochs, as is conventional [10,11,26].

The VO_2_ values were analysed in 10-second epochs for analysis, as suggested for the nature of the activities being performed [28]. Subsequently, VO_2_ were then converted into METs using the resting data where the children were lay supine. Estimated daily resting metabolic rate (RMR) was determined for each participant using the age, sex and mass specific Scholfield prediction equation [29], and METs were calculated by dividing energy expenditure by predicted (RMR). METs were then coded into one of four age-specific intensity categories (sedentary <1.5 METs), light (1.5–2.99 METs), moderate (3–5.99 METs) and vigorous (>6 METs), as per Harrell et al. [30]. Similar to the body of the literature on this topic [10,14] none of the activities resulted in MET values in excess of 6. Data were therefore utilised to represent 3 intensity categories reflecting sedentary, light and moderate PA (MPA).

### 2.4. Procedures (Cross-Validation)

For the cross-validation sample, the procedures employed are reported in full by Duncan et al. [14]. In brief, procedures for collection of breath by breath calorimetry and the use of GENEActiv accelerometers were identical to those reported above for the current study, other than the Duncan et al. [11] data set only used accelerometers placed on the dominant wrist, non-dominant wrist, waist and dominant ankle. The activities involved in the Duncan et al. [14] data set also differed slightly and comprised: lying seated and playing with Lego, slow walking (3 kph^−1^), medium-paced run (4.5 kph^−1^), a fast-paced run (6 kph^−1^), overarm throwing and catching a standard size tennis ball (20 passes per minute) and instep passing a football (size 3, 20 passes per minute). All activities were performed for 5 min, with a 5-minute rest in between.

### 2.5. Statistical Analysis

For the calibration, prior to analysis, data were checked for normality, which confirmed that data were non-normal via the Shapiro-Wilk test (all P < 0.05). Spearman’s rank correlations were therefore employed to examine criterion validity of the GENEActiv output at each wear location and METs. To determine SB and MPA cut-points, Receiver Operating Characteristic (ROC) curve analysis was undertaken [31]. The area under the curve (AUC) was calculated for each analysis, as a measure of diagnostic accuracy, as per guidelines [32] with AUC values of ≥0.90 considered excellent, 0.80–0.89 good, 0.70–0.79 fair, and <0.70 poor. ROC curve analysis was conducted as described previously [10,26] and cut-points that maximised sensitivity (Se) and specificity (Sp) were derived [33]. In line with prior work, AUC was determined for SB and MPA, leaving accelerometer counts that fell between the sedentary and MPA cut-points classified as light PA, in line with prior work [11]. Cut-points for light PA were classed as those higher than SB but lower than MPA but did not require AUC, Se or Sp values to be determined, as per Phillips et al. [11]. These are subsequently labelled as not applicable (NA) in Table 1 and Table 2. ROC analysis was undertaken using the Statistical Package for Social Sciences (SPSS, version 24). Recognising that this process determines cut-points related to PA intensity AUC was reanalysed, using the same process described above, to examine validity of GENEActiv output at each wear location to distinguish the FMS activity from the other activities. This resulted in cut-points representing FMS and non-FMS activity.

The cut-points developed in the calibration study were applied to the Duncan et al. [14] dataset and analysed using ROC curve analysis, using the same procedures as described above. In this way, we sought to compare how well the cut-points for children involving object control skills derived in the calibration could classify intensity of the activities used by Duncan et al. [14], which were different, but also included object control skills, and thus provide a cross-validation of our cut-points with an independent but comparable dataset. This cross-validation approach was undertaken using the cut-points that distinguished PA intensity (METS), and those that distinguished FMS to non-FMS activity.

## 3. Results

### 3.1. Calibration

**Figure 1:**
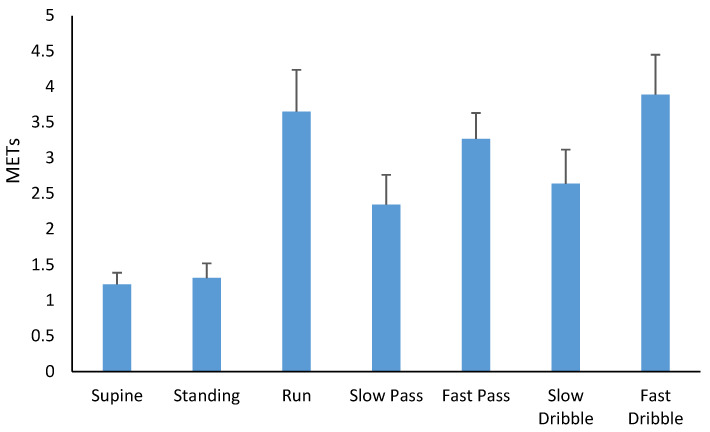
The mean ± SD of METs across each activity.

In regard to indirect calorimetry, being supine and standing were classified as sedentary (<1.5 METs), passing a football and dribbling a football at a slow cadence were classified as light (1.5–2.9 METs), while the medium-paced run, passing at a fast pace and dribbling at a fast pace were classified as moderate-intensity in nature (>3 METs). The mean ± SD of METs across each activity are presented in Figure 1.

The results from Spearman’s rank correlations indicated significant moderate relationships between METs, assessed using indirect calorimetry and GENEActiv counts at the dominant wrist (rho = 0.693, P = 0.0001), non-dominant wrist (rho = 0.673, P = 0.0001) and dominant ankle (rho = 0.702, P = 0.0001), and also between METs and GENEActiv counts at the waist (rho = 0.746, P = 0.0001) and non-dominant ankle (rho = 0.702, P = 0.0001).

The results for ROC curve analysis for GENEActiv accelerometers indicated that irrespective of wear location, they were able to successfully discriminate different intensities of activity. AUC values with 95% Confidence Intervals, values for sensitivity and specificity and resultant cut-points for each wear location are presented in Table 1.

ROC curve analysis indicated that irrespective of location, GENEActiv monitors were able to discriminate sedentary activity to an excellent level. Sensitivity and specificity values were similar for each wear location for sedentary behaviour. For moderate PA, the discrimination of activity was considered good for monitors placed on the dominant wrist, waist, non-dominant ankle and dominant ankle, while the discrimination of activity was only fair for the non-dominant wrist. The sensitivity and specificity values were also the poorest for the non-dominant wrist, with the best sensitivity and specificity values demonstrated for the dominant ankle in relation to MPA.

When data were reanalysed using ROC analysis to distinguish between FMS and non-FMS activity, the results indicated a fair discrimination for the dominant and non-dominant wrist, and poor discrimination for the waist and dominant and non-dominant ankles (See Table 2).

### 3.2. Cross-Validation

Table 3 presents the AUC, sensitivity and specificity of the cut-points derived in the current study, when applied to the data from the Duncan et al. [14] study to correctly distinguish the breath by breath derived MET values. ROC curve analysis indicated that the cut-points validated in the present study were able to successfully discriminate sedentary behaviour, light PA and moderate PA from the Duncan et al. [14] data set. AUC values were considered excellent for sedentary behaviour and moderate PA, whilst they were only considered fair for light PA.

When the cut-points determined in the calibration for FMS activity were applied to the cross-validation sample, examining their ability to discriminate between FMS and non-FMS activity in this independent data set, the AUC values were all considered poor with values for all placement locations <0.2. Such a result indicates that the cut-points offer little validity in discriminating FMS from non-FMS in the independent cross-validation sample.

## 4. Discussion

With the increasing use of accelerometery to assess SB and PA in children and young people, there has been a need for continued studies to examine how well accelerometers perform when classifying different types of PA. The present study contributes to understanding of this topic. Recent research has acknowledged the need to consider the ability of accelerometers to classify activity involving FMS. Recent work by Duncan et al. [14] examined how accelerometers classified throwing and catching and instep passing a football, while other work by Sacko et al. [24] identified that accelerometery was likely to classify object control skills as comprising light PA, when in fact they were moderate in nature. Sacko et al. [15] also identified that tempo of object control skill practice influenced the energy expended in FMS. The results of the present study extend this aforementioned work, by examining how effectively accelerometers classified key fundamental movement skills (football passing and dribbling) that relate to both PA and sport participation, when worn at five different locations of the body, and when these skills were performed at fast and low cadence.

The results of the present study identify that discrete skill practice of football passing and dribbling, when performed at a fast tempo, would be considered health-enhancing in terms of the metabolic costs of such activity. These fundamental movement skill activities were comparable to running at a medium pace on a treadmill. Importantly, these results confirm that such discrete skill practice could contribute to children meeting the recommended guidelines for health-enhancing physical activity. This is particularly relevant, given the latest recommendations for children’s PA, which emphasise the attainment of moderate-intensity PA and improving movement skills [17]. Studies investigating this issue have only recently emerged, which makes drawing comparisons more difficult. However, the results from the present study would agree with assertions made by Duncan et al. [14] that the practice of instep passing a football was considered moderate in intensity. The present results would also align with assertions made by Sacko et al. [24], based on adult data, that blocked practice of object control skills, including throwing, kicking and striking, resulted in metabolic expenditure that was moderate to vigorous in nature. In the Sacko et al. [14] study, children performed each FMS every 6–30 s with maximal force, whereas in the current study passing was performed 10 passes/min or 20 passes/min. This repetitive nature makes performance of the FMS in the current study is similar in nature to a serial skill such as rowing. This repetitive practice of skills thus results in health enhancing PA, whilst also helping to enhance skill performance. If performed in this way over time, not only is the activity of moderate intensity in nature, but it will also increase motor competence, which could leaded to increased confidence and competence to take part in a wider range of activity later in life, congruent with the tenants of the Stodden et al. [34] conceptual model. For Physical Education and Public Health practitioners, this is a key finding, which demonstrates that a focus on skill development can also be considered health-enhancing in terms of PA. It is important to note that the Stodden et al. [34] conceptual model posits that engagement in PA, particularly that of a moderate intensity and higher, can result in improvement in children’s motor competence. Likewise, engaging in activities designed to enhance motor competence can also lead to engagement in higher levels of PA [32]. This bidirectional PA–motor competence relationship underlines why it is important that activities such as FMS be considered when developing cut-points to better assess children’s PA intensity.

The GENEActiv accelerometers demonstrated acceptable criterion validity with METs across all five wear locations employed in the current study. Spearman’s correlations evidenced significant relationships between METs and GENEActiv counts irrespective of wear location, while the AUC analysis demonstrates that the GENEActiv output could accurately classify the criterion measure of MET values.

With regard to the accuracy of the accelerometers at different wear locations, the results from the calibration study suggest that accelerometer classification was excellent for sedentary behaviour and good for moderate PA. However, placement at the waist yielded the strongest AUC values for both sedentary behaviour and moderate PA. Recently, there have been suggestions that ankle-based accelerometer placement may be preferable to other locations in relation to the classification accuracy of PA [8,14]. The results of the present study suggest that this may not be the case, and that waist-based accelerometer placement results in better classification than wrist or ankle worn accelerometers. In the Duncan et al. [14] study, the authors asserted that the inclusion of football passing in their protocol may have been one reason why ankle worn accelerometers performed better. If this was the case, arguably, ankle worn accelerometer performance should have been markedly better in the current study, given the volume of lower leg movement required in the object control skills involved. Likewise, Duncan et al. [14] noted that the lack of standing as a discrete task in their study was a limitation. The current study addresses this issue and may also have contributed to the discrepancy in findings between the current study and that of Duncan et al. [14], as standing versus being seated will likely produce a different magnitude of acceleration in the ankle, whilst both activities are considered as sedentary.

The design employed in the current study is similar to that used by previous authors [10,11,14] in terms of calibrating accelerometer cut-points for PA intensities. Such an approach is valuable in being able to determine time spent in different intensities of PA. However, such an approach does not examine the applicability of accelerometers to assess FMS activities over non-FMS activities. In order to address this, we undertook secondary analysis using ROC analysis to determine if accelerometer output could distinguish between the two types of activity. The results from the calibration sample suggested that there was at best fair discrimination of FMS versus non-FMS activity from the accelerometer output. When applied to the cross-validation sample, the results suggest that the accelerometer output was not sufficiently able to distinguish between FMS and non-FMS activity. To some extent, this finding is not surprising. For example, the GENEActiv output for passing a football (considered FMS) and running (considered non-FMS) is more similar, than dissimilar, and the intensity of the activity is proportionate to the accelerometer output, hence why, in the calibration, fair to poor discrimination was evident. Likewise, there was no evidence that the FMS cut-points could discriminate this type of activity when applied to the cross-validation sample. Such a finding may be because the calibration sample included FMS activity comprising passing and dribbling a football, whereas the cross-validation sample included FMS activity comprising passing a football and throwing and catching. Thus, it is perhaps not surprising that the classification accuracy was poor when applied to the cross-validation sample when considered in this way. The movement patterns for executing a throw and catch are inherently different than dribbling a football, and while they may be similar in terms of PA intensity, they may differ in terms of the accelerometer output pattern they produce in isolation. The current findings therefore demonstrate the complexity of using sensor-based measures to assess individual human movements that are multiplanar and multidirectional. However, when considered in terms of the PA intensity that is required, irrespective of the movement type, the accelerometers are able to discriminate activity intensity.

One important facet of the present study is the use of a design which permitted cross-validation of the cut-points produced during the calibration using an independent sample. The current study involved secondary data analysis of the data reported by Duncan et al. [14], by applying our cut-points to this previous but comparable data. In this way, the current study addresses a noted limitation of accelerometer calibration studies, where such calibration studies do not then perform their own subsequent cross-validation [10,11,14]. The results of the cross-validation were performed for all wear locations other than the non-dominant ankle, as Duncan et al. [14] did not use this placement, and this suggests that the cut-points presented in this paper performed to a good standard in classifying sedentary behaviour and moderate-intensity PA in this separate sample. To some extent, this may have been anticipated as data collection processes were similar and the findings here and in that of Duncan et al. [14] included medium-paced running and instep passing a football, thus, there is some similarity in the activities involved. We are conscious that a matched, independent, data set comprising the same FMS activities undertaken in different samples was not available, and also that our calibration sample comprised boys only, whereas the cross-validation comprised boys and girls. Both of these points could be considered a limitation. However, by applying our cut-points to this independent data set we are able to cross-validate the cut-points presented in this manuscript on a different range of activities, which also included different FMS (throwing and catching). This is an appropriate means to establishing if the cut-points derived for intensity of PA are valid. As demonstrated, when attempts are made to distinguish between FMS and non-FMS activity, the results are less compelling. This may be a consequence of attempting to match two samples where different FMS are employed, or it may be because it is difficult to segregate an activity or moderate intensity which is FMS-based, compared to one that is not. The use of accelerometers to quantify time spent in different intensities of PA is relatively common, and our intention was to extend to this body of knowledge by incorporating FMS within such a protocol. Disentangling how accelerometers discriminate dribbling a football from, say, running at the same intensity of exercise using a single device worn on a single location is complex and beyond the scope of the current study. Further work, examining the possibility of combining locations together or using machine learning techniques, might be better placed to answer this question.

It is also important to note that this cross-validation only applies to the cut-points calibrated in the present study. The inclusion of FMS in the current study, and in the data set from the Duncan et al. [14] study provided the opportunity to compare against data using comparable, but different, activities, which both included FMS and both included monitors worn at the wrist, waist and ankle. Previously developed cut-points for children using the GENEActiv devices, such as those presented by Phillips et al. [11] have not included such activity or placement locations, instead using predominantly linear ambulatory movement in their calibration. As such, comparing the current data with those derived by Phillips et al. [11] may not be optimal. However, independent cross-validation of the previously derived cut-points [11,14] for use with this age of children alongside those presented here, may be of additional interest for researchers in the field. It could also be questioned whether additional cut-points are needed, as this may cause confusion for researchers and public health practitioners wishing to assess PA accurately using accelerometery. The current study has addressed a gap in the current range of cut-points, related to PA intensity, that are available for the GENEActiv for use in children by including FMS activity alongside the design of calibrating and cross-validating the cut-points in the same study. It should also be noted that the evolution of the understanding of children’s movement patterns and refinement of calibration processes will necessarily result in additional cut-points. A key next step is providing clear, evidence-based guidance for practitioners as to which cut-points might be most applicable, for which population and under which circumstances. Furthermore, while FMS are conceptualised as the foundation for PA [14,34] and are skills that are assumed to be prevalent during childhood, the children employed in the current study were considered to be regularly engaged in physical activity and had relatively ‘good’ motor competence. Therefore, the cut-points developed in the present study may not be wholly generalisable to children where there are deficiencies in motor development, or where object control motor skill has not been developed to a competent standard. The data presented in the current study are, however, based on laboratory-derived data, which likely means they are probably lower than occurs in free living. A next step is to apply these cut-points in free-living situations, particularly those which involve fundamental movement skills such as school physical education and community sports.

The current study extends the literature base in relation to children’s PA assessment by quantifying energy expenditure in object control-based FMS, executed at different cadences, as well as calibrating cut-points to distinguish sedentary, light and moderate physical activity in 8–12-year-old children. The results of the current study suggest that instep passing a football and dribbling a football at a cadence of 20 passes/min or 12 dribbles/min results in moderate-intensity energy expenditure that is considered health-enhancing. GENEActiv accelerometers demonstrated acceptable criterion validity, with waist-worn accelerometers providing the most suitable wear location to quantify moderate PA. The cut-points generated in the present study, when applied to an independent but comparable data set, also demonstrated a good ability to characterise sedentary behaviour and moderate-intensity PA, which involves the performance of fundamental movement skills.

## 5. Conclusions

Although the use of accelerometers to assess physical activity is becoming ubiquitous, the majority of prior studies fail to explore non-linear movement, such as those incorporating fundamental movement skills, in their calibration protocols. Even fewer cross-validate the accelerometer cut-points they derive on an independent sample. This study addresses these issues by providing cut-points for the assessment of children’s sedentary behaviour and moderate-intensity PA, including FMS, that are derived using breath by breath indirect calorimetry, and cross-validated in an independent sample, where data were derived in a similar manner. The derived cut-points presented here are therefore useful for those wishing to assess children’s movement, such as those working in Physical Education, public health and exercise science.

## Figures and Tables

**Table 1:** Area under the curve (AUC), sensitivity (%) and specificity (%) and resultant cut-points for each GENEActiv monitor with METs.

Intensity	Location	AUC	95% CI	Sensitivity	Specificity	Cut-Point g/s
**Sedentary**						
	Dominant Wrist	0.918	0.914–0.923	84.4	84.8	4.5
	Non-Dominant Wrist	0.913	0.909–0.918	84.7	84.6	4.4
	Waist	0.948	0.945–0.951	86.8	97.2	4.0
	Dominant Ankle	0.897	0.893–0.901	74.2	95.9	3.5
	Non-Dominant Ankle	0.911	0.907–0.915	82.5	96.4	4.0
**Light**						
	Dominant Wrist	NA	NA	NA	NA	4.6–16.4
	Non-Dominant Wrist	NA	NA	NA	NA	4.3–12.4
	Waist	NA	NA	NA	NA	4.1–8.8
	Dominant Ankle	NA	NA	NA	NA	3.6–39.2
	Non-Dominant Ankle	NA	NA	NA	NA	4.1–81.6
**Moderate**						
	Dominant Wrist	0.807	0.801–0.862	84.7	66.2	16.5
	Non-Dominant Wrist	0.791	0.786–0.797	67.5	53.3	12.5
	Waist	0.857	0.852–0.862	87.8	88.1	8.9
	Dominant Ankle	0.803	0.797–0.806	75.1	66.8	39.3
	Non-Dominant Ankle	0.849	0.843–0.854	76.3	81.2	81.7

**Table 2:** Area under the curve (AUC), sensitivity (%) and specificity (%) and resultant cut-points for each GENEActiv monitor with FMS activity.

Location	AUC	95% CI	Sensitivity	Specificity	Cut-Point g/s
Dominant Wrist	0.716	0.709–0.721	75.3	66.3	6.1
Non-Dominant Wrist	0.719	0.711–0.726	69.0	68.1	7.7
Waist	0.675	0.670–0.679	67.4	67.6	7.6
Dominant Ankle	0.665	0.659–0.670	65.4	66.7	35.1
Non-Dominant Ankle	0.622	0.615–0.926	59.1	68.1	31.8

**Table 3:** AUC, sensitivity and specificity of the cut-points derived in the current study, when applied to the data from the Duncan et al. [14] study to correctly distinguish the breath by breath derived MET values.

	AUC	95% CI	Sensitivity (%)	Specificity (%)
**Sedentary**				
Non-Dominant Wrist	0.924	0.914–0.934	87.9	89.0
Dominant Wrist	0.941	0.931–0.950	88.9	85.6
Waist	0.914	0.905–0.925	87.1	80.2
Dominant Ankle	0.907	0.896–0.919	87.1	76.6
**Light**				
Non-Dominant Wrist	0.791	0.771–0.812	62.0	64.8
Dominant Wrist	0.783	0.762–0.804	78.5	76.7
Waist	0.861	0.845–0.876	66.0	80.1
Dominant Ankle	0.754	0.738–0.771	63.1	83.9
**Moderate**				
Non-Dominant Wrist	0.954	0.945–0.963	66.9	86.1
Dominant Wrist	0.939	0.928–0.950	72.1	72.0
Waist	0.968	0.961–0.975	65.1	78.8
Dominant Ankle	0.921	0.912–0.931	65.2	74.3

## References

[1] Hallal P.C., Andersen L.B., Bull F.C., Guthold R., Haskell W., Ekelund U. (2012). Global physical activity levels: Surveillance progress, pitfalls, and prospects. Lancet.

[2] World Health Organization (2011). Global Recommendations on Physical Activity for Health 5–17 Years Old.

[3] US Department of Health and Human Services (2018). Physical Activity Guidelines Advisory Committee Scientific Report.

[4] Butte N.F., Watson K.B., Ridley K., Zakeri I.F., McMurray R.G., Pfeiffer K.A. (2017). A youth compendium of physical activities: Activity codes and metabolic intensities. Med. Sci. Sports Exerc..

[5] Kim Y., Beets M.W., Welk G.J. (2012). Everything you wanted to know about selecting the “right” Actigraph accelerometer cut-points for youth, but...: A systematic review. J. Sci Med. Sport.

[6] Newell K.M., Goodman D., Wilberg R.B., Franks I.M. (1985). Co-ordination, control and skill. Differing Perspectives in Motor Learning, Memory and Control.

[7] Rowlands A.V., Eston R.G. (2007). The measurement and interpretation of children’s physical activity. J. Sports Sci..

[8] Crouter S.E., Flynn Oody J., Bassett D.R. (2018). Estimating physical activity in youth using an ankle accelerometer. J. Sports Sci..

[9] Rowlands A., Rennie K., Kozarski R., Stanley R.M., Eston R.G., Parfitt G.C., Olds T.S. (2014). Children’s Physical Activity Assessed with Wrist- and Hip-Worn Accelerometers. Med. Sci. Sports Exerc..

[10] Roscoe C.M.P., James R.S., Duncan M.J. (2017). Calibration of GENEActiv accelerometer wrist cut-points for the assessment of physical activity intensity of preschool aged children. Eur. J. Ped..

[11] Phillips L.R., Parfitt G., Rowlands A.V. (2014). Calibration of the GENEA accelerometer for assessment of physical activity intensity in children. J. Sci. Med. Sport.

[12] Ryan J., Gormley J. (2013). An evaluation of energy expenditure estimation by three activity monitors. Eur. J. Sports Sci..

[13] Trost S.G., McIver K.L., Pate R.R. (2005). Conducting accelerometer-based activity assessment in field-based research. Med. Sci. Sports Exerc..

[14] Duncan M.J., Roscoe C., Faghy M., Tallis J., Eyre E. (2019). Estimating Physical Activity in Children Aged 8–11 Years Using Accelerometry: Contributions from Fundamental Movement Skills and Different Accelerometer Placements. Front. Physiol..

[15] Sacko R., McIver K., Brazendale K., Pfeifer C., Brian A., Nesbitt D., Stodden D. (2019). Comparison of indirect calorimetry and accelerometry-based energy expenditure during children’s discrete skill performance. Res. Q Exerc. Sport.

[16] Holfelder B., Schott N. (2014). Relationship of fundamental movement skills and physical activity in children and adolescents: A systematic review. Psych. Sport Exerc..

[17] Foster C. (2019). UK Chief Medical Officers’ Physical Activity Guidelines.

[18] Migueles J.H., Cadenas-Sanchez C., Ekelund U., Delisle Nystron C., Mora-Gonzalez J., Lof M., Labayen I., Ruiz J.R., Ortega F.B. (2017). Accelerometer data collection and processing criteria to assess physical activity and other outcomes: A systematic review and practical considerations. Sports Med..

[19] Montoye A.H., Moore R.W., Bowles H.R., Korycinski R., Pfeiffer K.A. (2016). Reporting accelerometer methods in physical activity intervention studies: A systematic review and recommendations for authors. Br. J. Sports Med..

[20] Bouten C.V., Westerterp K.R., Verduin M., Janssen J.D. (1994). Assessment of energy expenditure for physical activity using a triaxial accelerometer. Med. Sci. Sports Exerc..

[21] Westerterp K.R. (1999). Physical activity assessment with accelerometers. Int. J. Obes. Rel. Met. Disord..

[22] da Silva I.C., van Hees V.T., Ramires V.V., Knuth A.G., Bielemann R.M., Ekelund U., Brage S., Hallal P.C. (2014). Physical activity levels in three Brazilian birth cohorts as assessed with raw triaxial wrist accelerometry. Int. J. Epid..

[23] Clark C.C.T., Nobre G.C., Fernandes J.F.T., Moran J., Drury B., Mannini A., Gronek P., Podstawski R. (2018). Physical activity characterization: Does one site fit all?. Physiol. Meas..

[24] Sacko R.S., McIver K., Brian A., Stodden D.F. (2018). New insight for activity intensity relativity, metabolic expenditure during object projection skill performance. J. Sports Sci..

[25] Routen A., Upton D., Edwards M.G., Peters D. (2012). Discrepancies in accelerometer-measured physical activity in children due to cut-point non-equivalence and placement site. J. Sports Sci..

[26] Esliger D.W., Rowlands A.V., Hurst T.L., Catt M., Murray P., Eston R.G. (2011). Validation of the GENEA accelerometer. Med. Sci. Sports Exerc..

[27] Rowland T.W. (1995). Developmental Exercise Physiology.

[28] Mackintosh K.A., Ridley K., Stratton G., Ridgers N.D. (2016). Energy cost of free play activities in 10 to 11 year old children. J. Phys. Act. Health.

[29] Schofield W.N. (1985). Predicting basal metabolic rate, new standards and review of previous work. Hum. Nut. Clin. Nut..

[30] Harrell J., McMurray R., Bagget C., Pennell M.L., Pearce P.F., Bangdiwala S.I. (2005). Energy costs of physical activity in children and adolescents. Med. Sci. Sports Exerc..

[31] Jago R., Zakeri I., Baranowski T., Watson K. (2007). Decision boundaries and receiver operating characteristic curves: New methods for determining accelerometer cutpoints. J. Sports Sci..

[32] Metz C.E. (1978). Basic principles of ROC analysis. Seminars in Nuclear Medicine.

[33] Perkins N.J., Schisterman E.F. (2006). The inconsistency of “optimal” cutpoints obtained two criteria based on the receiver operating characteristic curve. Am. J. Epidemiol..

[34] Stodden D.F., Goodway J.D., Langendorfer S.J., Roberton M.A., Rudisill M.E., Garcia C., Garcia L.E. (2008). A developmental perspective on the role of motor skill competence in physical activity: An emergent relationship. Quest.

